# Robotic‐assisted approaches to urachal carcinoma: A comprehensive systematic review of the safety and efficacy outcomes

**DOI:** 10.1002/bco2.333

**Published:** 2024-02-01

**Authors:** Caio Vinícius Suartz, Lucas Motta Martinez, Pedro Henrique Brito, Carlos Victori Neto, Maurício Dener Cordeiro, Luiz Antonio Assan Botelho, Fábio Pescarmona Gallucci, José Maurício Mota, William Carlos Nahas, Leopoldo Alves Ribeiro‐Filho

**Affiliations:** ^1^ Urology Department, Hôpital Européen Georges Pompidou, AP‐HP. Centre Université Paris Cité Paris France; ^2^ Division of Urology, Institute of Cancer of São Paulo University of São Paulo São Paulo Brazil

**Keywords:** bladder cancer, partial cystectomy, robotic surgery, urachal adenocarcinoma, urachal carcinoma

## Abstract

**Introduction:**

Surgical intervention is the treatment of choice in patients with urachal carcinoma. Due to complications and to reduce hospital stay from open surgery, minimally invasive approaches are desirable. Nowadays, robotic‐assisted surgery has become increasingly popular, and robot‐assisted cystectomy can be performed in patients with urachal carcinoma with low complication rates.

**Methods:**

We performed a systematic review to search for studies that evaluated patients who underwent robotic‐assisted surgery for urachal carcinoma. The outcomes of interest were the type of cystectomy performed, whether there was umbilicus resection, total operative time, console time, intraoperative complications, estimated blood loss, postoperative complications, time of hospitalisation, positive surgical margins and the presence of documented tumour recurrence.

**Results:**

In this study, we evaluated three cohorts comprising a total of 21 patients. The median follow‐up period ranged from 8 to 40 months. Medium age was between 51 and 54 years, with a majority (63.1%) being male. One patient (5.2%) underwent a radical cystectomy, and 19 patients (94.7%) underwent to partial cystectomy. Umbilical resections were performed in all cases, and pelvic lymphadenectomy in 14 cases (73.6%). Recurrence occurred in three patients at a median of 17 months postoperation, two cases in the trocar insertion site. Additionally, there was one death, which was attributed to postoperative cardiovascular complications.

**Conclusion:**

Robotic‐assisted partial cystectomy has a low incidence of adverse outcomes in patients with urachal carcinoma. Controlled studies, ideally randomised, are warranted to establish the comparative efficacy and safety of the robotic‐assisted cystectomy approach relative to open surgery.

## 1 INTRODUCTION

Urachal carcinoma (UrC) is an uncommon neoplasm originating from the urachal remnant and represents less than 0.5% of all bladder neoplasms,^1^ with an incidence of less than one in 100.000 per year. The urachus, an embryonic remnant between the bladder dome and umbilicus, is the origin of UrC.^2^ The disease often presents a diagnostic and therapeutic challenge due to its rarity, leading to a dearth of extensive epidemiological studies and dedicated treatment protocols. Despite its infrequent occurrence, UrC demands attention owing to its aggressive nature and the distinct therapeutic approach it necessitates, diverging from typical urothelial cancers.^3^

Historically, the surgical management of UrC has predominantly involved open partial or radical cystectomy, often extending to the umbilicus or even further, as dictated by tumour extent.^4^, ^5^ Such procedures invariably involve a sizeable midline incision, carrying the associated morbidities of considerable blood loss, prolonged hospital stays and an extended recovery period.^6^ The search for improved surgical modalities, offering better postoperative outcomes without compromising oncological efficacy, has been perpetual.^7^

In this surgical landscape, robotic surgery has heralded a new era of promise.^8^, ^9^ Over the past two decades, robotic‐assisted techniques have revolutionised many aspects of urologic surgery, boasting potential advantages of enhanced precision, reduced morbidity and expedited recovery.^9^ Yet, the niche application of this technology in the realm of UrC is still nascent, with fragmented evidence and anecdotal experiences forming the bulk of our current understanding.

Recognising this knowledge gap, the present systematic review endeavours to amalgamate the scattered pieces of evidence. Our aim is to offer a comprehensive overview of the current state of robotic treatment for UrC, critically examining its efficacy, safety and outcomes.

## 2 EVIDENCE ACQUISITION

### 2.1 Literature search

The study was conducted in strict compliance with the Preferred Reporting Items for Systematic Reviews and Meta‐Analysis (PRISMA)^10^ statement on October 01, 2023. A research question was established based on the Patient‐Index test‐Comparator‐Outcome‐Study design (PICOS) criteria, which is as follows^8^: What is the current paradigm of robotic surgery for UrC?

The search strategy was as follows: (UrC OR urachal adenocarcinoma OR urachal cancer) AND (cystectomy OR cystoprostatectomy OR bladder resection OR partial cystectomy) AND (robotic OR da Vinci OR robotic‐assisted OR robotic‐assisted).

We searched the following databases up to October 2023:

1. Cochrane Central Register of Controlled Trials (CENTRAL) 2020, Issue 3, in the Cochrane Library;
2. MEDLINE via Ovid (from 1946);
3. Embase via Ovid (from 1974);
4. LILACS (Latin American and Caribbean Health Science Information database, from 1982);
5. Scopus, Elsevier's citation tool (from 2004);
6. Web of Science/Web of Knowledge (Clarivate and Thomson Reuters) (from 1900);
7. Education Resources Information Center (ERIC) (from 1966);

and the following trial registries:

1. ISRCTN registry (http://www.isrctn.com);
2. ClinicalTrials.gov (http://www.clinicaltrials.gov);
3. Australian New Zealand Clinical Trials Registry (http://www.anzctr.org.au);
4. World Health Organization International Clinical Trials Registry Platform (ICTRP) (apps.who.int/trialsearch/); and
5. EU Clinical Trials Register(http://www.clinicaltrialsregister.eu).

### 2.2 Study screening and selection

Two independent authors (Caio Vinicius SUARTZ, CVS, and Lucas Motta Martinez, LMM) screened all retrieved records. Discrepancies were resolved by discussion. The full text of the screened papers was selected if found relevant to the present review.

### 2.3 Selection criteria

We selected articles written in English describing research and studies in humans. There were no other restrictions based on study design or time of publication. Meeting abstracts, case reports, letters to the editor and editorials were excluded. When finding studies from the same institution with overlapping patients and outcomes, we considered only the most recent data. We also excluded previous literature reviews and studies mixing the data of different approaches (open, laparoscopic, and robotic) without specifying the robotic surgery outcomes.

### 2.4 Variables

We included the number of patients undergoing cystectomy in the study, the type of cystectomy performed, whether there was umbilicus resection, total operative time, console time, intraoperative complications, estimated blood loss, postoperative complications, time of hospitalisation, positive surgical margins, presence of documented tumour recurrence and median follow‐up. The primary outcome of our review is to assess and report complication rates, as well as oncological outcomes after robotic cystectomy to UrC.

### 2.5 Risk of bias assessment

Given the single‐arm nature of this study, without a comparison group, we utilised the Joanna Briggs Institute Critical Appraisal Checklist for Case Series to perform risk of bias assessment^11^ The Joanna Briggs Institute (JBI) is an international, membership‐based research and development organisation within the Faculty of Health Sciences at the University of Adelaide. The purpose of this appraisal is to assess the methodological quality of a study and to determine the extent to which a study has addressed the possibility of bias in its design, conduct, and analysis. The checklist consists of 10 questions ranging from criteria for inclusion, selections of participants, reported about demographic, clinical and follow‐up information and statistical analysis. The answers can be yes, no, unclear or not applicable. A response of ‘no’ to any of the questions negatively impacts the quality of the study.

## 3 EVIDENCE SYNTHESIS

### 3.1 Literature screening

**FIGURE 1:**
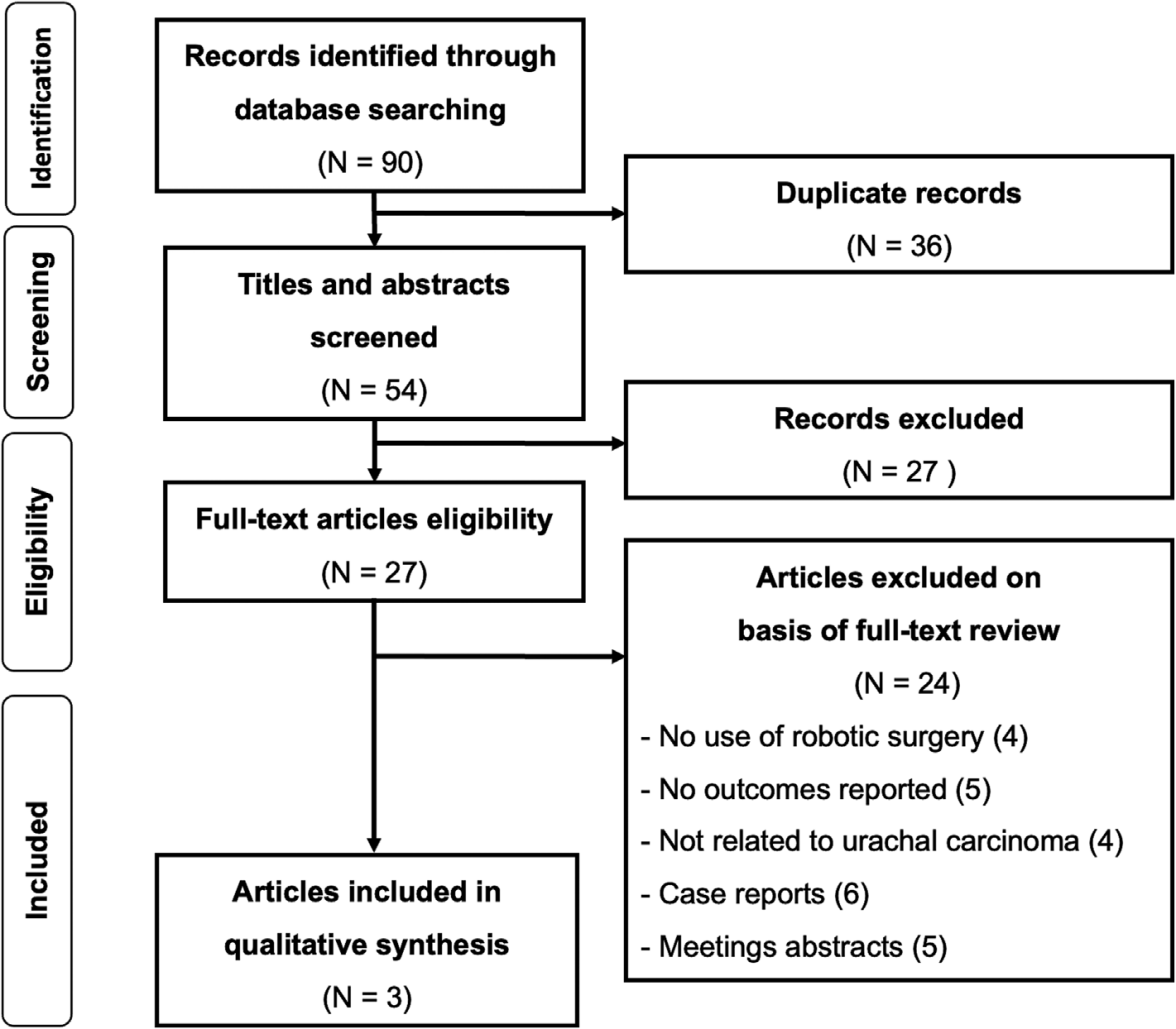
Preferred Reporting Items for Systematic Reviews and Meta‐Analysis (PRISMA) flowchart of the literature search.

The literature search retrieved 90 papers. Thirty‐six duplicate studies were automatically excluded. After the title and abstract screening of the remaining 54 unique references, 30 records were excluded because they were irrelevant to the study's aim. The full texts of the remaining 27 studies were assessed for eligibility. Finally, three studies were selected and included. Figure 1 shows the PRISMA flowchart of the literature search. We summarised in Table 1 information about the selected articles, such as institution, year of publication, country, patient gender, total number of patients and surgical technique utilised.

**TABLE 1:** Details of eligible studies.

Author	Year	Country	No. of patients	Gender (men/women)	Age	Technique	Pelvic lymphadenectomy	Umbilicus resection
Madeb et al.^12^	2006	United States of America	Three	2/1	Mean of 51 years SD (± 23.64)	Two Robotic Assisted Partial Cystectomy One Robotic Assisted Anterior Exenteration	Three patients	All cases
James et al.^13^	2015	United Kingdom	Eight	5/3	Mean of 53 years SD (± 10.3)	Eight Robotic Assisted Partial Cystectomy	Eight patients	All cases
Stokkel et al.^14^	2022	Netherlands	Eight	5/3	Median 60 years IQR (42–79)	Eight Robotic Assisted Partial Cystectomy	Three patients	All cases

Abbreviations: IQR, interquartile range; SD, standard deviation.

### 3.2 Quality assessment

The quality assessment of individual studies is reported in Table 2. All studies had a clear outline of the inclusion criteria for patient selection.

**TABLE 2:** Quality assessment of individual studies.

Author/year	Q1	Q2	Q3	Q4	Q5	Q6	Q7	Q8	Q9	Q10	Overall appraisal
Madeb et al. (2006)^12^	Yes	Yes	Yes	Yes	Yes	Yes	Yes	Yes	Yes	Yes	Included
James et al. (2015)^13^	Yes	Yes	Yes	Yes	Yes	Yes	Yes	Yes	Yes	Yes	Included
Stokkel et al. (2022)^14^	Yes	Yes	Yes	Yes	Yes	Yes	Yes	Yes	Yes	Yes	Included

### 3.3 Clinical and epidemiological characteristics

In a comparative series from the Netherlands, Stokkel (2022) evaluated the outcomes of robot‐assisted versus open partial cystectomy for UrC.^14^ The study spanned from 1994 to 2020, with eight patients undergoing the procedure, seven of whom had adenocarcinoma. Patients who underwent robotic surgery presented a median age of 60 years, with a range from 42 to 79 years, 5 men and 3 women.^14^

The Madeb study conducted between January 2005 and February 2006 included five patients with a median age of 51 years, ranging from 24 to 68 years. Two men and one woman with the anatomopathological revealing two well‐differentiated and one moderately adenocarcinoma of the urachus colonic subtype with margins and negative lymph nodes. Another two cases presented anatomopathological leiomyoma and squamous metaplasia in the urachus. The clinical presentations varied between gross hematuria and a normal physical examination. The computerised tomography revealed, in all cases, bladder mass in dome topography extending to the anterior wall without lymph node involvement.^12^

The James study, conducted from 2009 to 2014, consisted of five men and three women with a mean age of 53.5 years (±10.3 years). All patients presented with hematuria, dysuria or urinary mucus. Preoperative evaluation included MRI and CT scans with mass in the dome or anterior bladder wall. Cystoscopy with biopsy and colon preparation were utilised in all patients.^13^

### 3.4 Surgical technique

In the methodology proposed by the authors,^12^, ^13^, ^14^, the patient was placed in the Trendelenburg position to enhance the surgical exposure of the pelvic region. Flexible intraoperative cystoscopy was used to assist in resection for all patients, and just James mentioned the robotic platform utilized (Da Vinci S and SI robotic systems). All studies located the camera port between 2 and 5 cm cephalad to the umbilicus to facilitate both the umbilectomy, which was performed by a circumferential incision at a distance of 2 cm from the umbilicus, and the closure of the incision with the umbilicoplasty described by Schaeffer^15^ preserving the aesthetic aspect. Strategic placement of the camera port incision was prioritized to allow its subsequent incorporation into an umbilectomy incision, thereby reducing the necessity for additional incisions and associated surgical trauma. After the camera port placement, two 8‐mm robotic ports and one 12‐mm assistant port were inserted into the right iliac fossa. In certain instances, an additional trocar was employed to assist the procedure. The configuration of the port sites was planned to promote the ergonomic handling of instruments, ensure optimal articulation of the robotic arms, and facilitate surgical access to urachus with minimal aesthetic impact.

### 3.5 Perioperative considerations

In the series reported by Stokkel et al.,^14^, the established therapeutic approach for UrC involved partial cystectomy with En‐bloc excision of the urachal remnant and the umbilicus. All cases were performed with flexible cystoscope assistance, and the bladder was distended with 300 mL of saline to improve the visualization of robotic excision. The urachus was sectioned at the umbilical junction and pelvic lymphadenectomy was selectively performed based on tomographic or clinical evidence of nodal metastasis, with three out of eight patients (38%) undergoing this procedure. Resection of the umbilicus was universal among the cohort, and comprehensive preoperative staging was conducted using thoracoabdomino‐pelvic computerized tomography and bone scintigraphy prior to 2011, succeeded by fluorodeoxyglucose‐positron emission tomography (FDG‐PET) imaging. The treatment regimen incorporated neoadjuvant external beam radiation and brachytherapy targeting the bladder suture line. Postoperative care entailed a week of catheterization and cystography preceding catheter removal to ensure the absence of urinary leakage. Surveillance protocols were stratified by disease stage, with quarterly cystoscopies during the initial biennial period.^14^

Madeb's study emphasised that the positioning and placement of portals are fundamental to optimising the range of motion for the robotic arms, facilitating the complete resection of the urachus.^12^ The bladder was distended with 250–300 mL of saline to improve the visualisation to bladder resection and the urachus was sectioned at the umbilical junction. The dissection of the Retzius space revealed the anterior pelvic fascia, allowing bilateral pelvic lymphadenectomy and urachal excision with the umbilicus. The radical cystectomy procedure included the dissection of the ureters, ovarian and uterine arteries, and the block removal of the anterior vaginal wall and urethra. Average blood loss was reported as 118 m, with a range of 25–300 mL. The duration of the operative time was observed to vary from 120 to 300 min.^12^

The total exenteration of the anterior pelvis was completed in 480 min, followed by a six‐day postoperative hospitalisation. For those submitted to partial cystectomy, the discharge time varied from 36 to 48 h. Routine cystography was performed within 10 days after partial cystectomy, confirming the absence of urinary leaks.^12^

The study by James et al. utilised the Hason pneumoperitoneum method, utilising a six‐port array comparable to that in radical prostatectomy, which was modified by elevating the port sites by 3 cm to guarantee unobstructed access to the urachal region. The umbilicus was excised, aligning with the camera port positioned 2 cm above it. To enhance the surgical field, the bladder was inflated with 200 mL of air. A dual‐lens camera system, with zero and 30° angles, afforded clear visualisation for the bladder dissection and cystotomy, maintaining a 2‐cm margin from the identified bladder mass via integrated flexible cystoscopy. Bladder closure and drain insertion were performed in all selected studies with a double‐layered Vicryl 2‐0 suture and a Jackson–Pratt drain into the Retzius space.^13^

James et al. referred to an operative time average of 184 min, with a range of 130 to 240 min, and the mean duration of console operation of 120 min, ranging from 70 to 170 min.^13^ The estimated blood loss was minimal, averaging 50 mL, with no cases requiring conversion to open surgery. The average hospital stay was 4 days, with a range of 3 to 7 days. Drains were typically removed after 2.5 days. The cystography conducted at the 10‐day mark postoperatively confirmed the absence of leaks. The postoperative course was uncomplicated, with no instances of positive surgical margins.^13^

### 3.6 Oncological outcomes

In the Stokkel series, patients had a median follow‐up of 40 months and a median overall survival of 28 months.^14^ Local recurrence was observed in 15% of the cohort. The median time to recurrence was 21 months. One patient died of postoperative complications related to past medical history of cardiovascular disease that included an abdominal aortic prosthetic grafting. Anticoagulant therapy was discontinued prior to surgery, and a prophylactic dose of heparin was administered. After the surgery, the patient received brachytherapy with strict immobilisation for 3 days and ended up developing an acute arterial infrarenal occlusion of the aortic prosthetic graft 4 days after the surgery and died. There was only one case of persistent urinary leak for which the indwelling catheter was successfully left in place for a more extended time.^14^

Stokkel et al. described two port sites recurrences post robot‐assisted partial cystectomy. The author utilized positron emission tomography‐computed tomography (PET‐CT) with fluorodeoxyglucose (FDG) to identify an umbilical lesion at the anterior port site of the robotic trocar, which biopsy confirmed as a metastatic UrC tract.^14^

In the first case, subsequent to the FDG‐PET‐CT imaging, the patient underwent a secondary diagnostic laparoscopy. This revealed multiple peritoneal lesions proximal to the excised umbilicus, the site of UrC mobilisation during the initial surgery. The patient was treated with cytoreductive surgery and hyperthermic intraperitoneal chemotherapy (HIPEC). Seven months postsurgery, additional lesions were identified bilaterally at the lower abdomen anterior to the robotic‐assisted partial cystectomy trocar sites and at the median laparotomy site. Despite resection of all abdominal wall lesions, disease progression ensued, culminating in patient death after 10 months.

In the second case, the patient received adjuvant brachytherapy along the bladder suture lines. Although the initial resection was complete (R0), the patient experienced a local recurrence 2 years postoperatively. The recurrence was managed with external beam radiotherapy, local resection, and brachytherapy. A subsequent metastasis at the trocar site led to its local excision and further investigative diagnostic laparoscopy, which revealed advanced peritoneal carcinomatosis. Owing to the significant tumor load, additional cytoreductive surgery with HIPEC was deemed infeasible. The patient was administered palliative chemotherapy and died approximately 1 year following the diagnosis of peritoneal disease.

Kaplan–Meier analysis revealed no significant difference in recurrence‐free survival between patients undergoing robot‐assisted surgery and open partial cystectomy. Tumour sizes were predominantly under 4 cm, with most patients achieving negative surgical margins (88%). Lymphovascular invasion was identified in a single case. The study concluded that the 2‐year and 5‐year overall survival and recurrence‐free survival rates were comparable between the two surgical modalities.^14^

Madeb et al. observed a 100% recurrence‐free rate among their patient cohort over a median follow‐up period of 8 months without deaths.^12^ Similarly, James et al. reported no recurrences within an average follow‐up of 32 months without deaths.^13^

## 4 DISCUSSION

The studies conducted by Madeb (2006), James (2015) and Stokkel (2022) highlight the oncological efficacy and perioperative safety of robotic surgery as the minimally invasive treatment of choice for patients with UrC, improving surgical outcomes and reducing morbidity.^12^, ^13^, ^14^

Robotic‐assisted cystectomy remains the technique's principles of open surgery with complete excision of the urachus, umbilicus and pelvic lymphadenectomy^16^, ^17^ with the benefits of precise dissection and resection, minimal blood loss and reduced hospitalisation time.^12^, ^13^, ^14^, ^15^, ^16^ However, it is essential to manage the tumours carefully and to be cautious when removing the specimens, thus avoiding the implantation of tumour cells in the trocar sites, as evidenced by Stokkel.^14^

Mantica et al. (2020) highlight the potential underreporting of such recurrences, suggesting a publication bias that may skew data to centres reporting favourable results.^18^ This challenges the accuracy of reported incidence rates and raises concerns about the generalisation of results, reinforcing the importance of works such as that of Stokkel et al. when reporting in detail cases of recurrence in port sites.^14^

Progress in surgical proficiency, technique refinement, careful manipulation of the tumour and the containment of the urine leak in the moment of partial cystectomy need to be paramount in preserving tumour integrity and preventing the spillage of tumour cells in the abdominal cavity. Adopting techniques such as replacing saline solution to insufflation with air to facilitate bladder filling, as suggested by Peak and Hemal (2020), exemplifies some strategies that aim to minimise the chance of tumour recurrences.^19^

However, despite these advances, recurrences at the port site remain a nonnegligible risk. These cases, highlighted by the literature cited, reinforce the need for postoperative surveillance and establishing robust protocols for local control. Aggressive local control can be critical to management and potentially mitigate the impact of these relapses on patient outcomes.^14^, ^15^, ^16^, ^17^, ^18^, ^19^

Madeb (2006) and James (2015) have documented the evolution of robot‐assisted surgical techniques, with a notable decrease in positive surgical margins as surgeons move up the learning curve. This indicates improved surgical accuracy and suggests a potential for better oncological results.^12^, ^13^

However, a comparison between open, laparoscopic and robotic techniques is hampered by the need for more scientific research describing the outcomes in a comparative approach to patients with UrC. Currently, there is an absence of research on the robotic surgical technique employed relating it to clinical outcomes since most studies are case reports without technical details, perioperative and/or oncological outcomes. There are innumerable series of UrC that did not stratify patients by the surgical technique employed, analysing patients operated with open, laparoscopic and robotic approaches as a single group.

All studies used the Intuitive® robotic platform (da Vinci Si, X, or Xi), which reinforces the need for scientific validation of the new multiports and single ports robotic platforms to treat this specific group of patients.

The limited number of cases in these three series restricts the generalizability of the results. New studies individualising patients in the analysis by surgical technique are necessary to ensure the surgical safety and oncological efficacy of treatment with robotic assistance.

## 5 CONCLUSION

Robotic surgery represents a paradigm shift in the management of UrC potentially offering superior results. Robotic partial cystectomy in this clinical condition has a low incidence of adverse outcomes and can be done safely and effectively, but controlled studies, ideally randomised, are justified to establish the comparative efficacy and safety of the robotic‐assisted approach in relation to open surgery.

## AUTHOR CONTRIBUTIONS

**Caio Vinicius Suartz:** Idealization, data collection, analysis and writing of the manuscript. **Lucas Motta Martinez:** Data collection. **Pedro Henrique Brito:** Data collection. **Carlos Victori Neto:** Data collection. **Maurício Dener Cordeiro:** Supervision and correction of the manuscript. **Luiz Antonio Assan Botelho:** Correction of the manuscript. **Fábio Pescarmona Gallucci:** Correction of the manuscript. **José Maurício Mota:** Supervision and correction of the manuscript. **William Carlos Nahas:** Supervision and correction of the manuscript. **Leopoldo Alves Ribeiro‐Filho:** Idealization, supervision and correction of the manuscript.

## CONFLICT OF INTEREST STATEMENT

The authors declare no conflicts of interest.

## FINANCIAL SUPPORT

None.
